# Memories of Seven Campaigns

**Published:** 1898-12

**Authors:** 


**Memories of Seven Campaigns.** By JAMES HOWARD THORNTON, C.B., M.B. Pp. xxviii., 359. Westminster [London]: Archibald Constable and Co. 1895.

This is an interesting narrative of the adventures of Dr. Thornton during thirty-five years service in the Indian Medical Department.

The first indications of the great Sepoy Mutiny were becoming apparent when Dr. Thornton sailed for India in 1856; and it was not long after Dr. Thornton's arrival that the storm burst in Bengal. With the 5th Fusiliers he joined the column under the command of Major Vincent Eyre, and witnessed the defeat of the mutineers at Arrah. He was present at the relief and subsequent capture of Lucknow under Sir Colin Campbell. For two years he was employed with the troops in quelling the mutiny, which was terminated in 1859.

His second campaign was in China, where he was present at the siege and capture of the Taku Forts, at the surrender of Pekin, and the capture of the Summer Palace by the French and the Cavalry of our Indian Army.

The next important campaign in which Dr. Thornton was engaged was in the Soudan, after the destruction of the army under the command of Hicks Pasha. Dr. Thornton, volunteering for service in the Soudan, was appointed Principal Medical Officer of the troops, under the late General Sir John Hudson, the strength of which was 3,250 officers and men. The destination of the force was Suakin. This force was kept pretty busy, for the celebrated Osman Digna was in command of the enemy's forces. First came the battle of Tofrik, which was a surprise to the British force, and there was for a time considerable confusion: the enemy fought with desperate courage, but even their valour was of no avail against the destructive fire which was directed on them. The loss on the British side amounted in killed, wounded, and missing to 300 fighting men and 176 followers, while the Transport Service lost 500 camels and a number of mules. This chapter in the record of this hard-worked medical officer is very interesting and exciting. After a good deal of garrison work had been done at Suakin the war came completely to an end. Dr. Thornton obtained a well-earned leave of absence to England; and on arrival had the satisfaction to find that the dignity of the Companionship of the Order of the Bath had been conferred on him, in addition to which he received—published in General Orders—the thanks of the General Officer commanding the Indian Contingent. Without going into the details of other campaigns in India in which Dr. Thornton was engaged, we may say that the work of the medical officer of the Indian Service is no bed of roses. Dr. Thornton returned to India after the Suakin Campaign, was appointed Principal Medical Officer to the Frontier Field Force, and after a long and faithful service he retired with the rank of Deputy Surgeon-General.

The book is handsomely got up and is copiously illustrated.

